# Targeting CXCR4 in AML and ALL

**DOI:** 10.3389/fonc.2020.01672

**Published:** 2020-09-04

**Authors:** Daniel Cancilla, Michael P. Rettig, John F. DiPersio

**Affiliations:** Division of Oncology, Department of Medicine, Washington University School of Medicine, St. Louis, MO, United States

**Keywords:** AML, ALL, CXCR4, CXCL12, chemosensitization

## Abstract

The interaction of acute myeloid leukemia (AML) and acute lymphoblastic leukemia (ALL) blasts with the bone marrow microenvironment regulates self-renewal, growth signaling, as well as chemotherapy resistance. The chemokine receptor, CXC receptor 4 (CXCR4), with its ligand chemokine ligand 12 (CXCL12), plays a key role in the survival and migration of normal and malignant stem cells to the bone marrow. High expression of CXCR4 on AML and ALL blasts has been shown to be a predictor of poor prognosis for these diseases. Several small molecule inhibitors, short peptides, antibodies, and antibody drug conjugates have been developed for the purposes of more effective targeting and killing of malignant cells expressing CXCR4. In this review we will discuss recent results and strategies in targeting CXCR4 with these agents in patients with AML or ALL.

## Introduction

**Figure 1:**
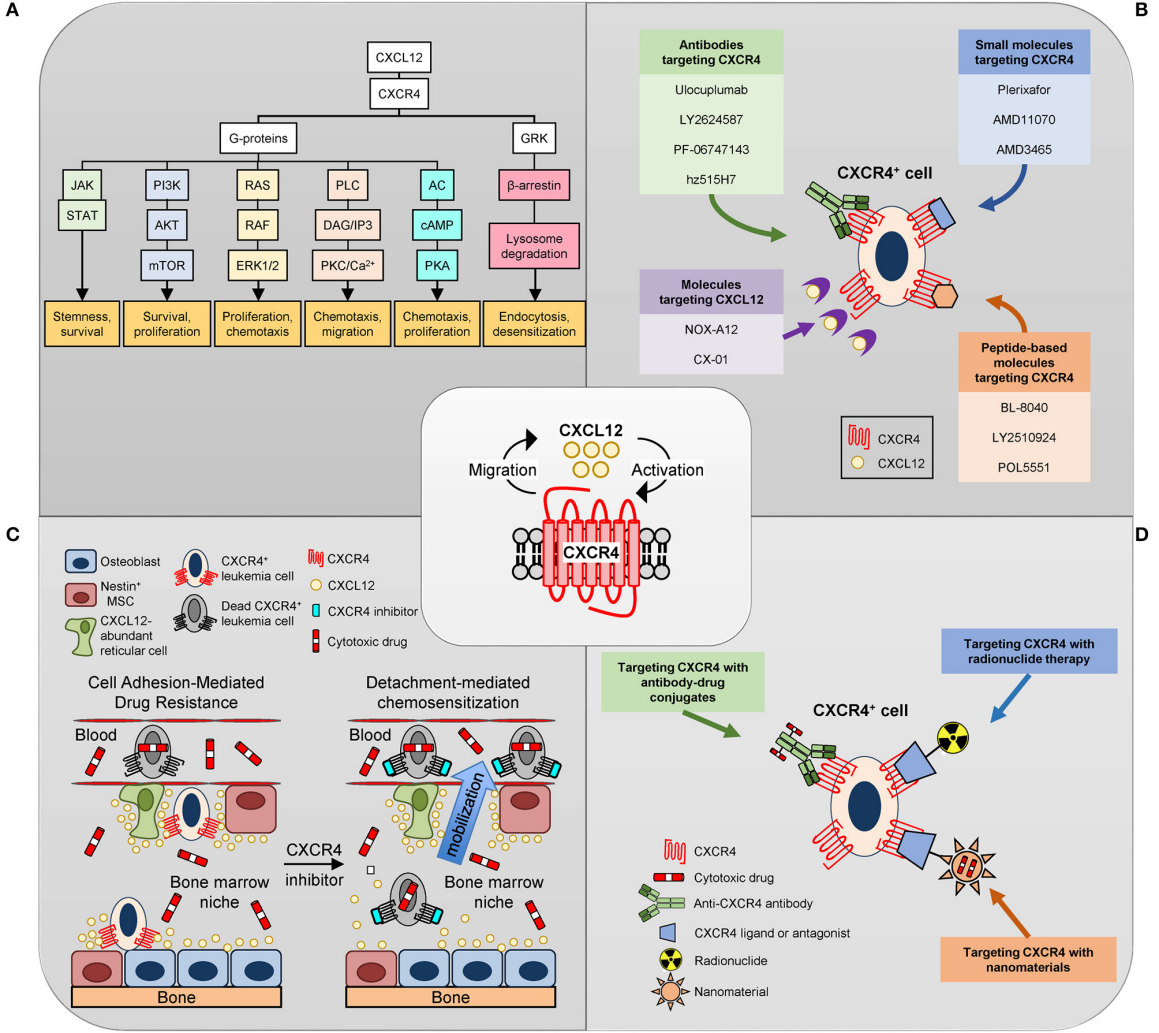
Targeting CXCR4. **(A)** Overview of CXCL12/CXCR4 signal transduction pathways. CXCR4 is a seven-transmembrane spanning G-protein coupled receptor. Upon binding CXCL12, CXCR4 G-protein-mediated signaling leads to (a) activation of JAK/STAT signaling promoting stemness and survival, (b) activation of the phosphatidylinositol-3-kinase (PI3K)-Akt-mTOR pathway promoting cell survival and proliferation, (c) activation of Ras/Raf and mitogen-activated protein kinase (MAPK) signaling leading to phosphorylation of extracellular signal-regulated kinase (ERK) 1/2 which promotes survival and proliferation, (d) activation of phospholipase C (PLC) leading to diacylglycerol (DAG) and inositol triphosphate (IP3) production which induces Ca^2+^ efflux and protein kinase C (PKC) activation resulting in chemotaxis and migration, and (e) inhibition of adenylyl cyclase (AC), which reduces cAMP production and protein kinase A activation leading to reduced cell proliferation and chemotaxis. CXCL12 also induces CXCR4 desensitization via uncoupling the receptor from G-proteins by GRK-dependent phosphorylation and subsequent β-arrestin-dependent endocytosis and lysosomal degradation. **(B)** Inhibitors of the CXCR4/CXCL12 axis. Drugs targeting CXCR4 can be divided into three general categories: (1) small molecules; (2) peptide-like molecules; and (3) antibodies. NOX-A12 disrupts the CXCR4/CXCL12 axis by binding and neutralizing CXCL12. **(C)** Schematic of CXCR4 inhibitor-mediated chemosensitization in leukemia. Osteoblasts, mesenchymal stem/stromal cells (MSC) and CXCL12-abundant reticular (CAR) cells in the bone marrow niche constitutively express CXCR12 leading to the recruitment and adhesion of CXCR4-expressing leukemia cells. These interactions provide a protective environment to the malignant cells in the presence of chemotherapeutic agents. Disruption of CXCR4-mediated adhesion of leukemia cells to the chemoprotective cells in the bone marrow niche via administration of CXCR4 inhibitors promotes mobilization and chemosensitization of leukemia cells. **(D)** Alternative approaches to target CXCR4-expressing cells. Antibodies to CXCR4 have been linked to cytotoxic drugs (antibody-drug conjugates; ADC) which induce cell death via DNA damage and/or microtubule disruption upon internalization. Several small molecule inhibitors and peptide-based antagonists of CXCR4 have been radiolabeled for positron emission tomography (PET) imaging or endoradiotherapy. Nanomaterials are small particles that serve as carriers for small-molecule drugs, proteins, and nucleic acids. Several nanomaterials targeting CXCR4 with the small molecule inhibitors, antibodies, or peptide-based antagonists discussed above have been developed.

The chemokine receptor 4 (CXCR4) is a member of the G protein coupled receptor (GPCR) family of receptors and plays a role in numerous biological processes including fetal organ development, hematopoiesis, and immune system function. It is expressed on a wide range of hematopoietic cells including hematopoietic stem and progenitor cells (HSPCs) T cells, B cells, monocytes, macrophages, eosinophils, and neutrophils (1). The CXCR4 receptor was initially discovered in 1996 and studied in the context of human immunodeficiency virus-1 (HIV-1) as a co-receptor for viral entry into the cell (2). Further research revealed the role of CXCR4 in chemotaxis of white blood cells, retention of HSCs in the bone marrow, as well as involvement in several signaling pathways important for cellular proliferation, survival, and chemotactic migration [Figure 1A; for review see (3, 4)]. The CXCR4 receptor interacts with the peptide signaling molecule stromal cell-derived factor 1 (SDF-1), also known as CXCL12, which is produced by bone marrow endothelial and stromal cells, including CXCL12-abundant reticular (CAR) cells (5–7). The migration of CXCR4 expressing cells along the CXCL12 gradient contributes to the chemotaxis of these cells and their retention within the bone marrow niche (7). The pro-survival effects of the CXCR4-CXCL12 interaction are thought to be mediated by activating mammalian target of rapamycin (mTOR) and downstream translation, while increased cellular proliferation is induced via activation of the signaling molecule extracellular signal related kinase (ERK-1/2) (3, 4).

CXCR4 is highly expressed in over 20 cancer types, ranging from hematological malignancies to solid tumors (8, 9). In leukemia, the receptor contributes to the retention of malignant cells in the bone marrow, which is thought to offer a protective environment to these cells in the presence of chemotherapeutic agents. This has been shown across multiple cancer types including acute myeloid leukemia (AML) and acute lymphoblastic leukemia (ALL) (10, 11). Therefore, CXCR4 might be an attractive molecular target for treating these malignancies. One predominant rationale for targeting the CXCL12/CXCR4 axis in AML and ALL is that its disruption will not only inhibit pro-survival signaling but also mobilize the leukemic cells from the protective bone marrow environment and out into the vasculature where they might be more susceptible to conventional chemotherapeutic agents (Figures 1B,C). This strategy is termed “chemosensitization” and has been tested in both animal models and human trials.

Recent studies have shown that in addition to being able to mobilize leukemic cells to the vasculature, some CXCR4 inhibitors have direct cytotoxic effects and can induce apoptosis in cells expressing the receptor. Because of these properties, there is growing interest in using CXCR4 inhibitors as part of an ablative chemotherapy regiment in the setting of hematopoietic cell transplant (HCT) or even as a monotherapy (12). Several small molecule inhibitors, short peptides, antibodies, and antibody drug conjugates (ADC) have been developed for the purposes of more effective targeting and killing of malignant cells expressing CXCR4 (13, 14). In this review we will discuss recent results and strategies in targeting CXCR4 with these agents in patients with AML or ALL.

## Aml

Acute myeloid leukemia (AML) is a clinically heterogeneous malignancy. A primary characteristic of this disease is the elevated level of immature myeloblasts in the marrow or peripheral blood. It is the leading cause of acute leukemia in the adult population and accounts for more deaths than any other leukemia (15). The current prognosis for patients with AML is poor, with a 5-year survival rate of only 24% (16). Up to 40% of patients with AML fail to respond to initial therapy and remain refractory to all available treatment regimens from outset (17). In general, for relapsed or refractory patients with AML (rrAML), therapeutic choices are limited and the prognosis is exceptionally bleak, with a median overall survival of about 4 months (18). There is no established standard of care for patients with rrAML (18). Allogeneic hematopoietic cell transplant (HCT) is regarded as the only strategy with any possibility of a meaningful period of disease control but is limited by significant transplant-related morbidity and mortality associated with high doses of chemotherapy and radiation therapy, which are needed to prepare patients for transplant, as well as graft-vs.-host disease (GvHD) (19).

## All

Acute lymphoblastic leukemia is a hematologic malignancy characterized by impaired differentiation and proliferation of lymphoid progenitor cells. The age adjusted incidence of ALL is ~1.7 per 100,00 per year, with higher incidence in children than adults. ALL can be divided into B-cell ALL and T-cell ALL, with the former making up approximately 75% of cases and the latter making up ~25% of cases (20). While treatments for ALL in children are very effective with cure rates around 90%, treatments in adults are much less effective (21). Despite improved treatments, including the emergence of bispecific antibodies, chimeric antigen receptor therapy, and tyrosine kinase inhibitors for Philadelphia chromosome positive ALL, the overall adult survival rate is <45% (22). Furthermore, in adults 60 years or older the 5 year survival rate is <20% (23). Across all ages, relapse affects over 30% of patients. Relapsed or refractory ALL is associated with particularly poor outcomes with a reported 10% 3 year survival rate in B-cell ALL. Outcomes are even worse in T-cell ALL (24). There is a clear need for additional therapies that can reduce the rate of relapse in these patients.

## CXCR4 Expression and AML/ALL Prognosis

Studies have shown that increased expression of CXCR4 is associated with poor outcomes in patients with AML and B-cell ALL. This is potentially due to the role CXCR4 plays in keeping leukemic cells within the bone marrow as well as its roles in activating pathways that favor the survival, growth, and chemotherapy resistance of these malignant cells (1). Leukemic blasts with high CXCR4 expression in cases of AML was associated with higher rates of relapse and lower overall survival (25–35). Patients with a FMS-like tyrosine kinase-3 internal tandem duplication (FLT3-ITD) or nucleophosmin mutation express significantly more CXCR4 than their wild-type counterparts (27–29, 31, 32, 36). However, these cytogenetic abnormalities do not solely account for the elevated CXCR4 expression in the disease as high CXCR4 expression is still a risk factor for prognosis in normal karyotype patients with AML (29, 30). Further, CXCR4 in French American British (FAB) AML-M4 and M5 subtypes is significantly increased relative to the other FAB and World Health Organization (WHO) AML subtypes (27–29, 31).

Fewer studies have evaluated CXCR4 expression in ALL. Like AML, high expression of CXCR4 is associated with worse overall survival in patients with B-cell ALL (37–41). Although no reports have linked high CXCR4 expression with a poor prognosis in T-cell ALL, it has been shown that T cell ALL interaction with CXCL12 is required for maintenance of disease in mouse models and that T ALL cells from both humans and mice show higher CXCR4 expression when compared to healthy cells (42, 43). There is also evidence that both AML and ALL cells upregulate CXCR4 during treatment with chemotherapy, suggesting that CXCR4 may play a role in chemotherapy resistance (44–46). Taken together, these data suggest that the CXCL12/CXCR4 axis influences outcomes and therapy responsiveness in AML and ALL.

## CXCR4 Inhibition for Chemotherapy Sensitization

Given the body of evidence demonstrating that CXCR4 is not only a prognostic marker but also plays a role in leukemia progression, several strategies have emerged to potentially treat AML and ALL via targeting CXCR4. While the BM microenvironment supports proper development of hematopoietic stem and progenitor cells (HSPCs), it can also serve as a shelter for malignant cells by shielding them from cytotoxic chemotherapy (Figure 1C). It has been proposed that there is a sub-population of leukemic cells in AML and ALL know as leukemia initiating stem cells (LSC) that reside within the protective niche of the bone marrow (47–50). These cells are thought to be powerful contributors to drug resistance and relapse in these diseases. Chemosensitization aims to eliminate this LSC pool of cells by disrupting the interactions that keep them anchored within the bone marrow and exposing them to cytotoxic agents (10, 51, 52). In addition to disrupting the anchorage of the LSC pool within the bone marrow niche, a secondary effect of targeting CXCR4 is to disrupt the survival and growth signaling mediated by this receptor. This is evidenced by studies showing elimination of cancer cells with CXCR4 inhibitor monotherapy (53, 54). Therefore, the combination of cell detachment and inhibition of pro-survival signaling may contribute to the efficacy of CXCR4 targeting agents.

Pathways responsible for the anchorage and survival of malignant cells, and therefore for their therapy resistance, were found to largely overlap with those described for normal HSPCs [reviewed in (55–61)]. Numerous studies have shown that CXCR4 antagonism, defined as the disruption of the interaction between CXCR4 and CXCL12, leads to mobilization of HSPCs into the blood circulation. These observations taken together provide the underlying rational for disruption of the CXCR4/CXCL12 axis in an attempt to sensitize the malignant cell populations within the bone marrow. Multiple pre-clinical studies in mouse models of AML (12, 62–66) or ALL (43, 45, 67–71) have been reviewed elsewhere (10, 42, 51, 72) and provided proof of principle for the beneficial effects of combining CXCR4 inhibition with conventional chemotherapy compared to treatment with chemotherapy alone.

CXCR4 targeting drugs can be divided into four categories: (1) small molecule CXCR4 antagonists; (2) peptide-like CXCR4 antagonist; (3) antibodies to CXCR4; and (4) CXCL12 antagonists (Figure 1B). In general, both small molecule and peptide-like inhibitors of CXCR4 contain cationic regions capable of binding the predominantly anionic extracellular region of CXCR4. Crystal structures of CXCR4 complexed with a small molecule (isotiourea-1t; IT1t), peptide-like antagonist (CVX15) and a chemokine-like molecule (viral macrophage inflammatory protein 2) have been reported (73, 74). Not surprisingly, the peptide-like molecule mimicked the chemokine-like molecule in binding a major pocket in CXCR4 that is comprised of transmembrane (TM) domains three and seven of CXCR4 as well as more buried residues within the protein. In contrast, the IT1t small molecule bound a minor pocket between TM1-TM3 and TM7 in CXCR4. Subsequent site-directed mutational studies with different small molecule inhibitors have demonstrated that they can bind either the minor and/or major pockets of CXCR4 [for review see (75, 76)]. Below we discuss the clinical results published to date with these four classes of CXCR4 targeting drugs in patients with AML and ALL (Table 1).

**Table 1:** Clinical trials targeting CXCR4 in combination with chemotherapy in AML and ALL.

**CXCR4 Inhibitor**	**Combined regimens**	**Disease**	**Phase**	**Age of enrollment**	**ClinicalTrials.gov identifier**	**References**
Plerixafor	Mitoxantrone + Etoposide + Cytarabine	AML	1/2	18–70	NCT00512252	(77)
Plerixafor	Etoposide + Cytarabine	AML, B-ALL, MDS	1	3–29 (Pediatric)	NCT01319864	(78)
Plerixafor	Daunorubicin + Cytarabine	AML	1	18–70	NCT00990054	(54)
Plerixafor	Decitabine	AML	1	60+	NCT01352650	(79)
Plerixafor	Clofarabine	AML	1/2	60+	NCT01160354	-
Plerixafor	G-CSF + Mitoxantrone + Etoposide + Cytarabine	AML	1/2	18–70	NCT00906945	(80)
Plerixafor	G-CSF + Daunorubicin + Cytarabine	AML	1	18–65	EudraCT number 2011-000474-56	(81)
Plerixafor	G-CSF + Sorafenib	AML	1/2	18+	NCT00943943	(82)
Plerixafor	G-CSF + Fludarabine, Idarubicin, and Cytarabine	AML	1/2	18–65	NCT01435343	(83)
-	G-CSF + Isofamide + Etoposide + Dexamethasone	B-ALL, T-ALL	1	18+	NCT01331590	(84)
BL-8040	Cytarabine	AML	1	18–75	NCT01838395	(85)
BL-8040	Cytarabine	AML	2	18–75	NCT02502968	-
BL8040	Nelarabine	T-ALL	2	18+	NCT02763384	(86)
LY2510924	Idarubicin + Cytarabine	AML	1	18–70	NCT02652871	(87)
Ulocuplumab	Mitoxantrone + Etoposide + Cytarabine	AML	1	18+	NCT01120457	(88)
Ulocuplumab	Cytarabine	AML	1	18+	NCT02305563	-
CX-01	Idarubicin + Cytarabine	AML	1	18–80	NCT02056782	(89)
CX-01	Idarubicin + Cytarabine	AML		60+	NCT02873338	(90)
CX-01	Azacitidine	AML	1	18+	NCT02995655	(91)

### Chemosensitization With Small Molecule CXCR4 Antagonist Plerixafor

An extensive array of over 20 different chemical classes of small molecule CXCR4 antagonists have been developed since the original report of CXCR4 serving as a coreceptor for human immunodeficiency virus (HIV) 1 infection [for review (76)]. In 2008, the small molecule AMD3100 (plerixafor) was approved as an HSPC mobilizer (92–94). Clinically, plerixafor, in combination with granulocyte colony-stimulating factor (G-CSF), has proven effective for the mobilization of hematopoietic stem cells in patients with multiple myeloma (MM) and non-Hodgkin's lymphoma (NHL) undergoing autologous stem cell transplantation (53). Further, plerixafor has been tested as a chemosensitizing agent in patients with AML or ALL. Below we briefly discuss these trials, where patients were treated with plerixafor alone or in combination with G-CSF in an attempt to enhance the efficacy of cytotoxic chemotherapy.

#### Chemosensitization With Plerixafor Alone

In the initial phase I/II study using a small molecule CXCR4 antagonist as a chemosensitizing agent (NCT00512252), 52 patients with relapsed or refractory AML (rrAML) were administered plerixafor followed by Mitoxantrone + Etoposide + Cytarabine (MEC) chemotherapy for 5 days (77). Like HSPCs, the mobilization of AML blasts by plerixafor was transient, and cell counts returned to baseline within 12 h. Overall, 46% of the patients achieved a complete remission (CR) or complete remission with incomplete blood count recovery (CRi), which is a significantly higher rate compared to the historical response rate of 21% in similar patients receiving MEC chemotherapy alone (95). However, the median overall survival was only 8.2 months with a relapse-free survival of 9 months. Of note, the bolus injection of plerixafor in this trial induced significant upregulation of surface CXCR4 on AML blasts and increased their CXCL12-mediated chemotaxis. Since high CXCR4 expression is a marker of poor prognosis in AML, the plerixafor-mediated upregulation of CXCR4 on AML blasts might have reduced the efficacy of MEC chemotherapy in this trial.

In a small phase one trial of pediatric leukemia, 19 patients with relapsed or refractory leukemia (13 with AML, five with B-ALL) or myelodysplastic syndrome (MDS; *N* = 1) were treated with plerixafor for 5 days followed 4 h later by high dose cytarabine and etoposide (NCT01319864). This treatment regimen was well-tolerated although there was only a modest 17% (3/18) clinical response in three patients with AML (78). No responses were observed in patients with ALL or MDS.

In subsequent trials, the efficacy of plerixafor chemosensitization was evaluated in newly diagnosed patients with AML treated with (1) a combination of cytarabine and daunorubicin (7 + 3 regimen), (2) decitabine, or (3) clofarabine. In the first trial, 23 patients received cytarabine on days 1–7, daunorubicin on days 1–3, and plerixafor on days 2–7 (NCT00990054). With this regimen, which was similar in toxicity to chemotherapy alone, 67% of patients (14/21) demonstrated complete remission (54). In the second trial (NCT01352650), 69 elderly patients received monthly cycles of a 10 day decitabine regimen with plerixafor administered 4 h prior to decitabine during alternating treatment cycles (79). Plerixafor failed to effectively sensitize the AML blasts to decitabine chemotherapy with patients exhibiting an overall response rate of 43% that was similar to the 47% CR rate achieved in historical controls receiving decitabine alone (96). In the third trial (NCT01160354), plerixafor was administered to elderly patients (*N* = 22) 4–6 h prior to clofarabine for 5 consecutive days and no outcome data has been published to date.

#### Chemosensitization With Plerixafor Plus G-CSF

Since G-CSF acts synergistically when combined with plerixafor for HSPC mobilization (97, 98), it was proposed that this combination would more effectively disrupt AML blasts from the bone marrow microenvironment and render them susceptible to MEC chemotherapy. This hypothesis was further supported by previous studies indicating that “priming” with G-CSF prior to chemotherapy resulted in superior outcomes for patients receiving induction chemotherapy for AML (99). In the first chemosensitization trial with plerixafor, 20 patients with rrAML were treated with G-CSF (days 1–8), plerixafor (days 3–8) and MEC chemotherapy (days 4–8) (80). This study was terminated after an interim analysis revealed that only 30% (6 out of 20) of patients achieved a response with a median overall survival of 7.6 months (NCT00906945). In the second study, Heiblig et al. (81) tested a G-CSF (days 1–10) plus plerixafor (days 1–3 and 8–10) mobilization regimen in combination with daunorubicin (days 1–3), and cytarabine (days 1–3 and 8–10) in ten patients with AML after their first relapse from standard (7 + 3) induction chemotherapy (EudraCT number 2011-000474-56). Encouragingly, eight of nine evaluable patients (88%) achieved a response (5-CR; 3-CRi) and seven proceeded to an allogeneic HSCT. This increased response rate compared to the first combination trial might have been due to the enrollment of younger patients with a more favorable risk stratification (majority of patients were favorable or intermediate risk). In the third study, sorafenib (days 1–28) was tested in combination with G-CSF and plerixafor (every other day from days 1–13) in 33 patients with rrAML with FLT3-ITD mutations (NCT00943943). A complete response rate of 28% was observed in 21 evaluable patients, including three patients refractory to previous FLT3 inhibitors (82). Finally, 57 patients with rrAML were administered both G-CSF and plerixafor in combination with fludarabine, idarubicin, and cytarabine (NCT01435343). Here, fludarabine, cytarabine, G-CSF, and plerixafor were all administered on days 1–4, while idarubicin was only given on days 1–3 (83). The overall response rate of 49% (median overall and disease free survival of 9.9 and 13 months, respectively) was similar to a historical control group treated without plerixafor (100).

In contrast to AML, no reports to date describe ALL patients treated with plerixafor and G-CSF as part of a chemosensitization trial. However, 13 patients with rrALL (11 B-ALL; 2 T-ALL) were treated with G-CSF in combination with a salvage chemotherapy regimen consisting of isofamide with mesna, etoposide, and dexamethasone (NCT01331590). Three patients (2 B-ALL; 1 T-ALL) achieved a complete remission (CR/CRi) for an overall response rate of 23% (84).

### Chemosensitization With Peptide-Based CXCR4 Antagonists

BL-8040 is a 14 residue synthetic peptide that has a high affinity (1 nM) and a slow dissociation rate (>24 h) from CXCR4 (101). Abraham et al. (102) demonstrated that BL-8040 directly caused AML cells to undergo apoptosis both *in vitro* and *in vivo* using various mouse models. In contrast, plerixafor alone did not elicit the same type of cytotoxic effects as BL-8040 (103). In a recently completed phase 2a trial (NCT01838395), 42 patients with rrAML were treated with BL-8040 monotherapy for 2 days followed by combined administration of BL-8040 and high dose cytarabine (HiDAC) for 5 days over one to two cycles (85). The response rate for all dosing levels was 29% (12/42) with a median overall survival of 9.1 months that was >6.1 month median survival observed for a historical control group treated with HiDAC alone (104). In a separate trial (NCT02502968), patients with AML in first CR are treated with BL-8040 or placebo and HiDAC as part of a consolidation therapy approach. No data has been reported to date for this trial. Finally, in an ongoing trial involving patients with rrT-ALL, BL-8040 is being administered in combination with nelarabine (NCT02763384). Five of nine patients enrolled in the study have achieved a complete remission for an overall response rate of 56% (86).

LY2510924 is a small cyclic peptide containing non-natural amino acids that potently inhibits the binding of CXCL12 to CXCR4 (IC_50_ = 0.08 nM) and exhibits a long half-life (9.16 h) in humans (105). In a recently completed phase I study (NCT02652871), 11 patients with rrAML were treated with LY2510924 monotherapy for 7 days followed by combined administration of LY2510924 with idarubicin and cytarabine for 3 or 4 days (87). The overall response rate of 36% (4/11) was similar to a historical control group treated with idarubicin and cytarabine alone (106). One interesting observation from this trial was that, unlike plerixafor (77), LY2510924 administration was not associated with an upregulation of surface CXCR4 expression on the AML blasts (87). This lack of CXCR4 upregulation should prevent rapid re-homing of AML blasts to the protective BM microenvironment and promote the pro-apoptotic properties of CXCR4 blockade.

### Chemosensitization With Antibodies to CXCR4

At least four monoclonal antibodies (mAbs) against CXCR4 (Ulocuplumab, LY2624587, PF-06747143, and hz515H7) have been tested in humans and many more antibodies, nanobodies, and other fragments targeting the CXCR4/CXCL12 axis are in pre-clinical development [for review see Bobkov et al. (107)]. Compared to small molecule inhibitors and peptide-based antagonists, mAbs exhibit longer blood half-lives (up to several weeks for IgG) and, depending on the IgG subclass, can possess Fc domain-mediated effector functions that facilitate the elimination of target-expressing cells via antibody-dependent cell-mediated toxicity (ADCC), antibody-dependent cellular phagocytosis (ADCP), and/or complement-dependent cytotoxicity.

Ulocuplumab, an IgG4 mAb that inhibits the binding of CXCL12 to CXCR4 and induces caspase-independent apoptosis on multiple cell lines and primary samples from patients with AML, chronic lymphocytic leukemia (CLL), and multiple myeloma (108–110). A phase one trial in 73 adults with rrAML tested the antibody alone and in combination with MEC chemotherapy (NCT01120457). A 51% overall CR/CRi rate was observed in the 43 patients who received ulocuplumab in combination with MEC chemotherapy (88). Four patients achieved a CR/CRi upon treatment with BMS-936564 alone indicating the antibody has some single-agent anti-leukemia activity (88). A phase 1/2 study testing the antibody in combination with low dose cytarabine in newly diagnosed patients with AML was completed in June 2019 (NCT02305563) but no data has been published to date.

### Chemosensitization With Inhibitors of CXCL12

CXCL12 antagonists have been designed for the purposes of disrupting the CXCR4/CXCL12 axis and sensitizing malignant cells to chemotherapy. CX-01 (dociparstat sodium; DSTAT) is a low molecular weight derivative of heparin that binds CXCL12 and is able to neutralize PF4, an inhibitor of megakaryopoiesis (111–113). In a small trial of 12 patients with AML (NCT02056782), CX-01 (days 1–7) was administered alongside cytarabine (days 1–7) and idarubicin (days 1–3) as part of induction and consolidation chemotherapy. The treatment regimen was safe and well-tolerated, with no CX-01-associated serious events (89). An overall response rate of 92% (11/12) was attained with all of the responders presenting with *de novo* AML. The 92% CR rate is higher than a 71% CR rate observed in a historical control cohort treated with cytarabine and idarubicin (114). A follow-up randomized, dose-response phase two study (NCT02873338) of the same regimen in 66 older patients (>59 years old) with AML recently reported a similar CR rate of 89% as well as significantly improved event free survival (*P* = 0.019) when compared to standard treatment alone (90). In a separate trial, hypomethylating agent (HMA)-refractory (received four or more cycles of HMA without response or disease progression on HMA therapy) patients with AML or MDS were treated with a 7 day continuous infusion of CX-01 and azacitidine in 28 day cycles (NCT02995655). The median overall response rate in 15 evaluable patients was 27% (4/15) with a median overall survival of 221 days (91). A randomized Phase three trial evaluating CX-01 in combination with standard induction chemotherapy in newly diagnosed patients with AML is planned.

NOX-A12 is a pegylated L-enantiomeric RNA oligoribonucleotide (Spiegelmer) that binds and neutralizes CXCL12 thereby blocking its interaction with CXCR4 and CXCR7 (115, 116). In a phase one trial with healthy volunteers (NCT01194934), NOX-A12 was well-tolerated and mobilized CD34+ HSPCs in dose-dependent manner (116). Peak HSPC mobilization occurred within 1–4 h of NOX-A12 administration and, in accordance with the plasma-half-life of 38 h, remained elevated at 4 days after treatment. Although not tested in patients with AML and ALL to date, encouraging results have been obtained in clinical trials evaluating NOX-A12 in combination with bendamustine and rituximab for the treatment of relapsed or refractory patients with CLL (NCT01486797) (117) or bortezomib and dexamethasone in relapsed or refractory patients with multiple myeloma (NCT01521522) (118).

### Summary of Chemosensitization Studies Targeting CXCR4 in Patients With AML and ALL

The initial clinical trials with plerixafor, BL-8040, Ly2510924, and ulocuplumab discussed above have demonstrated the feasibility and safety of combining CXCR4 inhibitors with chemotherapy in patients with AML or ALL and provided *in vivo* evidence for disruption of the CXCR4/CXCL12 signaling axis. However, results from these studies have been disappointing and, taken together, there is no obvious benefit over chemotherapy alone. This is highlighted by the fact that none of the regimens have entered into randomized phase three trials. The phase 2b study assessing the safety and efficacy of BL-8040 vs. placebo as part of a cytarabine-based consolidation chemotherapy regimen in patients with AML is ongoing (NCT02502968) and the capability of BL-8040 to directly induce apoptosis in malignant but not normal cells may enhance the efficacy of chemosensitization. More favorable clinical responses have been observed following treatment of *de novo* AML patients with the CXCL12 antagonist CX-01 and chemotherapy. A randomized Phase three trial evaluating CX-01 in combination with standard induction chemotherapy in newly diagnosed patients with AML is planned. Since CXCR4 inhibitors haven't been tested in the upfront setting, it's unclear if the enhanced clinical activity observed with CX-01 is due to its different mechanism of action or the patient population under study.

## Using CXCR4 Inhibitors in Myeloablative Chemotherapy Regimens for HCT

All of the clinical studies discussed thus far have combined disruption of the CXCR4/CXCL12 axis with drug and/or chemotherapy regimens that were of insufficient intensity to ablate the recipient's hematopoietic system. Hematopoietic stem cell transplantation (HSCT) allows for treatment with myeloablative therapy without concern for harming normal HSCs because of the hematopoietic rescue mediated by the transplanted untreated (i.e., non-chemotherapy-exposed) donor HSCs. HSCT thus provides a platform to combine CXCR4 antagonism with myeloablative chemotherapy. The rationale for this approach is that CXCR4 inhibition is thought to enhance donor HSPC engraftment during myeloablative conditioning by disrupting recipient HSPC retention within the bone marrow and sensitizing the recipient's normal HSPCs and leukemia stem cells to the cytotoxic conditioning chemotherapy. Below we discuss three trials where plerixafor was used as part of a myeloablative conditioning regimen for HCT in pediatric and adult patients with AML (Table 2). To our knowledge no patients with ALL have been treated with this approach to date.

**Table 2:** Clinical trials targeting CXCR4 in myeloablative chemotherapy regimens for HCT.

**CXCR4 Inhibitor**	**Combined regimens**	**Disease**	**Phase**	**ClinicalTrials.gov identifier**	**References**
Plerixafor	G-CSF + Busulfan + Fludarabine	AML, MDS, CML	1/2	NCT00822770	(119)
Plerixafor	Busulfan + Fludarabine + TBI	AML	1/2	NCT01141543	(120)
Plerixafor	Fludarabine + Thiotepa + Melphalan + rATG	AML	1	NCT01068301	(121)

*TBI, total body irradiation; rATG, rabbit antithymocyte globulin*.

In the first trial, plerixafor and G-CSF were included as part of a busulfan and fludarabine conditioning regimen for 45 adult patients (34 AML, seven MDS, and four CML) undergoing allogeneic HSCT (NCT00822770) (119). Although there was no observed difference in overall survival when compared to historical datasets of patients receiving only busulfan + fludarabine treatment, patients who received the mobilizing drugs showed increased rates of myeloid chimerism and lower rates of graft vs. host disease (GvHD) (119). In follow-up correlative studies, a negative correlation between survival and the percentage of AML blasts before and after conditioning was reported (122). The second trial included 12 adults with AML in first remission who underwent allogeneic HCT after receiving conditioning with plerixafor, busulfan, fludarabine, and 400 cGy total body irradiation (NCT01141543) (120). Plerixafor was administered 6 h before fludarabine and busulfan for up to 4 consecutive days (days−5 to −2 pre-HCT). Of the 12 patients enrolled, only two experienced disease relapse post-transplantation and six were alive at a median follow-up of 67 months (120). In the third trial, plerixafor was included as part of a myeloablative conditioning regimen for 12 pediatric patients with rrAML undergoing a second allogeneic HSCT (NCT01068301) (121). With a median duration of follow-up of 332 days, four of the 12 patients (33%) were alive with two being disease free. Taken together, these studies demonstrate the safety and feasibility of adding plerixafor to a myeloablative conditioning regimen for allogeneic HSCT and further investigation is warranted.

## Using CXCR4 Inhibitors to Enhance Donor Engraftment After HCT

Plerixafor has also been tested for its ability to enhance donor engraftment and hematopoietic recovery after allogeneic HCT. Following myeloablative conditioning, post-transplant treatment with plerixafor led to increased recovery of donor-derived cells in a murine transplant model (123). However, this effect was not observed by others following non-myeloablative conditioning of mice (124). In a phase I/II trial (NCT1280955), 30 patients with high-risk hematologic malignancies (*N* = 11 patients with ALL; *N* = 15 patients with AML/MDS) receiving myeloablative conditioning were treated with plerixafor every other day beginning day +2 until day +21 or until neutrophil recovery after allogeneic HCT (125). Plerixafor treatment had minor effects, with treated patients recovering absolute platelet counts >20,000/μL only 1 day sooner than untreated patients. There was no difference in the time to neutrophil recovery between plerixafor-treated and untreated patients. This study demonstrated that plerixafor can be administered safely following myeloablative HCT but has minimal effects on enhancing hematopoietic recovery when given every other day at a dose of 240 μg/kg.

## Targeting CXCR4 with Antibody-Drug Conjugates

An antibody-drug conjugate (ADC) is a monoclonal antibody (mAb) conjugated to a cytotoxic drug via a chemical linker (Figure 1D). Upon binding a target antigen on the surface of a cell, the ADC complex is internalized and degraded resulting in the release of the cytotoxic payload and cell death. Two publications have described ADCs targeting CXCR4 (13, 126). Costa et al. (126) screened multiple CXCR4-targeted ADCs and demonstrated efficient killing of multiple AML and ALL cell lines *in vitro* and non-small cell lung cancer cells *in vivo*. Their optimal ADC, ADC 713, was a low-affinity humanized IgG1 antibody with reduced Fc-mediator effector function. Tolerability and safety studies performed in a human CXCR4 knock-in mouse model (127) revealed significant decreases in the numbers of circulating neutrophils and monocytes 3 days after the third injection of ADC 713. Although the numbers of bone marrow lineage-negative/Sca-positive/Kit-positive (LSK) cells and HSPCs were unchanged, there was a significant decrease in granulocyte-monocyte progenitors, suggesting some hematopoietic toxicity. Therefore, although CXCR4 may not be an ideal target for an ADC due to its widespread expression on normal HSPCs, mature leukocytes, kidney tubular epithelium and the adrenal gland (128), the majority of these cell populations are largely quiescent and should be relatively insensitive to auristatin-mediated cell killing.

## Targeting CXCR4 With Radionuclide Therapy

Several small molecule inhibitors and peptide-based antagonists of CXCR4 have been radiolabeled for positron emission tomography (PET) imaging [for review see (129, 130)]. Three of these compounds: (1) ^64^Cu-AMD3100 (131), (2) ^68^Ga-NOTA-NFB (132), and (3) ^68^Ga-pentixafor (133) have been tested in humans. The rapid renal excretion and low non-specific background accumulation of the high affinity ^68^Ga-pentixafor compound has driven its clinical development as a diagnostic agent for CXCR4 in hematological and solid cancers as well as inflammatory conditions (129). A proof-of-concept study in 10 patients with AML demonstrated variable expression of CXCR4, with five of ten patients exhibiting ^68^Ga-pentixafor uptake that correlated well with AML infiltration as determined by magnetic resonance imaging (134). These data suggest that *in vivo* imaging of CXCR4 expression in patients with AML is feasible using ^68^Ga-pentixafor.

Pentixather is an analog of pentixafor that allows linkage of beta emitting radionuclides (^177^Lu; ^90^Y) that are routinely used in clinical practice for various cancer radiotherapies (135, 136). Habringer at al. (137) recently reported that endoradiotherapy with ^177^Lu-pentixather effectively targeted CXCR4+ tumor cells and significantly reduced leukemic burden in patient-derived and cell-line-based models of T-ALL and AML. Subsequent first-in-human studies of CXCR4-directed endoradiotherapy with ^177^Lu-pentixather or ^90^Y-pentixather in 22 patients with hematological malignancies (includes four patients with AML) have demonstrated the feasibility of this approach when combined with high-dose chemotherapy as part of a conditioning regimen for autologous or allogeneic stem cell transplantation (137, 138). Patients became anemic, neutropenic and thrombocytopenic after treatment with ^177^Lu-pentixather or ^90^Y-pentixather (138). This hematological toxicity was expected as there is a significant “cross-fire” effect from the radiolabeled pentixather that kills healthy hematopoietic HSPCs within the bone marrow upon targeting CXCR4-expressing cells. Although increasing the potential for off-target toxicity, this cross-fire irradiation may prove beneficial as it eliminates the need to target every single malignant cell and could disrupt and/or eradicate the tumor-supporting niche (137). Importantly, platelet and neutrophil engraftment after HSPC transplantation were not impaired by the CXCR4-directed endoradiotherapy. Therefore, the bone marrow niche was capable of supporting normal HSPCs after ^177^Lu-pentixather or ^90^Y-pentixather treatment. Finally, new scaffolds for delivering radiation to CXCR4 for imaging and endoradiotherapy have recently been developed from the small molecule CXCR4 inhibitor LY2510924 (139, 140).

## Targeting CXCR4 with Nanomaterials

Nanomaterials are small particles that serve as carriers for small-molecule drugs, proteins, and nucleic acids. Non-targeted liposomes containing conventional chemotherapeutic agents exhibit decreased toxicity compared to their corresponding free drugs and have been approved for use in patients with AML or ALL. Several pre-clinical studies using nanomaterials targeted to CXCR4 with the small molecule inhibitors, antibodies, or peptide-based antagonists discussed above have been reported [see Wang et al. (141) for review]. Although none of these pre-clinical studies have tested the efficacy of the CXCR4-targeted nanomaterials against ALL cells to date, a couple of nanomaterial-based approaches have been tested against AML. First, Wang et al. (142) used CXCR4-targeted polymeric nanoparticles containing plerixafor to deliver siRNA against the transcription factor RUNX1 as well as inhibit the CXCR4-CXCL12 axis in a mouse model of AML. Second, Diaz et al. (143) generated a CXCR4-targeted nanoparticle (via T22 peptide) containing ricin that was internalized and promoted AML killing in a CXCR4-dependent manner (143). In general, the combined capability to block the CXCR4/CXCL12 axis (via CXCR4 targeting moiety) while simultaneously delivering a drug payload (via nanomaterial) with a CXCR4-targeted nanomaterial could potentially enhance the efficacy and safety profile of therapeutics against AML and ALL. Like CXCR4-targeted ADCs and endoradiotherapy the main concern with CXCR4-targeted nanomaterials is on-target, off-tumor toxicity to normal cell subsets.

## Targeting CXCR4 in Combination with Immune Checkpoint Blockade

BL-8040 is being tested in combination with the anti-PD-L1 antibody atezolizumab in intermediate and high-risk patients with AML who have achieved a CR following induction and consolidation therapy (NCT03154827). The rationale behind this combination is based on the observation across several studies that high CXCR4 expression is associated with decreased immune activation and diminished response to checkpoint inhibitor treatment (144, 145). The primary endpoint of this study is to assess whether the combination prolongs relapse-free survival. In addition to AML, the immune-mediated effects of BL-8040 are being examined in combination with PD-1/PD-L1 immune checkpoint inhibitors in several clinical studies involving solid tumors (pancreatic, gastric, non-small cell lung cancer). These early-phase studies are being driven by pre-clinical data demonstrating that CXCR4 antagonism can enhance the efficacy of immune checkpoint treatment [see Gorbet et al. (146) for review].

## Conclusion and Future Perspective

The CXCR4 receptor plays a crucial role in the maintenance of leukemia through a variety of mechanisms, including pro-survival signaling and keeping leukemia cells sequestered in the protective bone marrow niche. This, coupled with its high level of expression on more than 20 different types of cancers, including AML and ALL, make it an attractive target for treatment of these malignancies. To date, the predominant strategy used to target CXCR4 in patients with AML or ALL involves disruption of the CXCR4/CXCL12 axis in combination with cytotoxic chemotherapy.

Since the majority of these studies tested different chemotherapy regimens and enrolled small numbers of patients with different baseline characteristics, it's difficult to draw definitive conclusions about their relative effectiveness. In general, the results from these trials have been disappointing and only CX-01 is progressing toward a phase three trial.

Correlative studies from the chemosensitization trials discussed above have demonstrated that surface expression of functional CXCR4 often increases following inhibition of the CXCR4/CXCL12 axis. This upregulation of CXCR4 on the leukemic blasts may enhance pro-survival signaling and re-homing of the leukemic blasts to the protective BM niche thereby negating the efficacy of cytotoxic chemotherapy. One potential chemosensitization strategy that warrants further investigation is combining cytotoxic chemotherapy with a new class of CXCR4 inhibitors that are capable of biased antagonism of CXCR4. The peptide X4-2-6, which is derived from the second transmembrane helix and first extracellular loop of CXCR4, and small molecule CXCR4 inhibitor SEN071 have been shown *in vitro* to selectively inhibit the G protein signaling dependent chemotaxis mediated via CXCR4 but not the beta arrestin recruitment and subsequent receptor endocytosis (147–149). Inhibition of the arrestin mediated endocytosis pathway is a proposed mechanism of tolerance to CXCR4 antagonists such as plerixafor (148). Agents such as X4-2-6 and SEN071 that inhibit G protein signaling of CXCR4 while sparing arrestin function may have the potential to significantly increase the efficacy of CXCR4 mediated chemosensitization.

Recently, Ramakrishnan et al. (150) demonstrated that CXCR4 alone provides sufficient signaling in the absence of CXCL12 ligation to promote the expansion and survival of murine AML cells *in vivo*. Therefore, if CXCR4 expression is maintained, disruption of the CXCR4/CXL12 axis alone might be insufficient to effectively sensitize leukemic cells to cytotoxic chemotherapy. If CXCL12 is indeed dispensable for the growth and persistence of leukemic blasts (or a subpopulation of LSCs) in patients with AML and ALL, more effective chemosensitization might only be attained following concomitant inhibition of both CXCL12 ligation (via a CXCR4/CXCL12 antagonist) and CXCR4 signaling. Several inhibitors of the RAS-RAF-MEK-ERK and PI3K-AKT-mTOR pro-survival signaling pathways downstream of CXCR4 exist and could be tested [for review see (151–154)]. Alternatively, inhibition of downstream CXCR4 signaling could be achieved via downregulation of the receptor. Some previously reported strategies to downregulate CXCR4 include treatment with (i) the small molecule bromodomain and extra-terminal domain-containing (BET) proteolysis-targeting chimera (PRTOAC) ARV-825 (155), (ii) lenalidomide or pomalidomide (156), and (iii) small interfering RNA [for review see Wang et al. (141)]. Additional pre-clinical studies combining cytotoxic chemotherapy with inhibition of CXCL12 ligation (via a CXCR4/CXCL12 inhibitor) and CXCR4 signaling or expression are warranted.

Finally, novel methods of mobilizing HSPCs continue to be studied with a number of pre-clinical compounds and/or regimens under investigation [for review see (157–159)]. Like healthy HSPCs, AML and ALL blasts express molecules other than CXCR4 that mediate adherence to BM stromal cells. Further, these molecules, such as VLA-4, LFA-1, E-selectin, and CD44, have been shown to provide anti-apoptotic and anti-proliferative effects and mediate chemotherapy resistance. Two phase three clinical trials testing the effectiveness of GMI-1271, an E-selectin inhibitor, to chemosensitize AML blasts in patients with *de novo* (NCT03701308) and rrAML (NCT03616470) are ongoing. Simultaneously targeting CXCR4 and these other adhesive interactions may increase the efficacy of chemotherapy sensitization.

## Author Contributions

DC, MR, and JD contributed to the writing of the manuscript. All authors contributed to the article and approved the submitted version.

## Conflict of Interest

MR and JD have pending patent applications (PCT/US2017/059777; Integrin inhibitors and chemokine receptor agents, and PCT/US2017/059733 Integrin Antagonists) and report royalties received from them. JD is founder and advisor for Magenta Therapeutics, receives income, has equity ownership, and has received research funding from them. JD is an Advisory Board Member for Cellworks Group. BiolineRx has provided research funding for the work in the JD and MR laboratories. The remaining author declares that the research was conducted in the absence of any commercial or financial relationships that could be construed as a potential conflict of interest.
